# 1690. Acute Hematogenous Osteoarticular Infections in Children at Siriraj Hospital, Thailand: A 20-Year Retrospective Review

**DOI:** 10.1093/ofid/ofad500.1523

**Published:** 2023-11-27

**Authors:** nichkamol lertamornkitti, orasri Wittawatmongkol, Kulkanya Chokephaibulkit

**Affiliations:** Siriraj Hospital, bangkok, Krung Thep, Thailand; Siriraj Hospital, bangkok, Krung Thep, Thailand; Siriraj hospital/Mahidol University, Bangkok, Krung Thep, Thailand

## Abstract

**Background:**

Acute hematogenous osteomyelitis (AHO) and septic arthritis (SA) lead to significant morbidity and sequelae. There are limited data in Thai children.

**Methods:**

This is a single-center retrospective study of patients aged < 18 years who were diagnosed with AHO and SA between 2002- 2021. The acute onset of AHO and SA was defined as the diagnosis of bone and joint infection within 4 weeks after the onset of symptoms or signs in a previously uninfected bone. Data were collected and factors associated with sequelae were determined by using multiple logistic regression analyses.

**Results:**

Of the 110 patients identified, 19 were AHO, 81 were SA, and 10 were combined AHO and SA. The median (IQR) age was 6.7 (1.4-11.9) years and 66 (60%) were male. Fifteen (13.6%) patients were immunocompromised. The most common sites of AHO were tibia (27.6%) and femur (27.6%), and of SA were hips (41.8%) and knees (31.9%). The pathogens were identified in 68 of 106 (64.1%) patients with specimen submission. The positivity rate of hemoculture, synovial fluid, synovial tissues, bone biopsies and vaginal/urethral culture in patients with suspected disseminated gonococcal infection were 32.1%, 40%, 61.9%, 60% and 50% respectively. The most common organisms were methicillin-sensitive *Staphylococcus aureus* (30.2%) and non-typhoidal *Salmonella* (9.4%). We found only one case with community-acquired, methicillin-resistant *S.aureus*. Non-typhoidal *Salmonella* was responsible for 22.7% of 22 infants. The one-year sequelae were found in 15.8% of AHO, 18.5% of SA, and 20% of combined. In multivariate models, factors associated with sequelae were being immunocompromised (adjusted OR 14.16, 95%CI 1.96-102.35, P=0.009) and infection of the hips (adjusted OR 6.25, 95%CI 1.45-14.59, P=0.025).

Patients' characteristics

**Figure:**
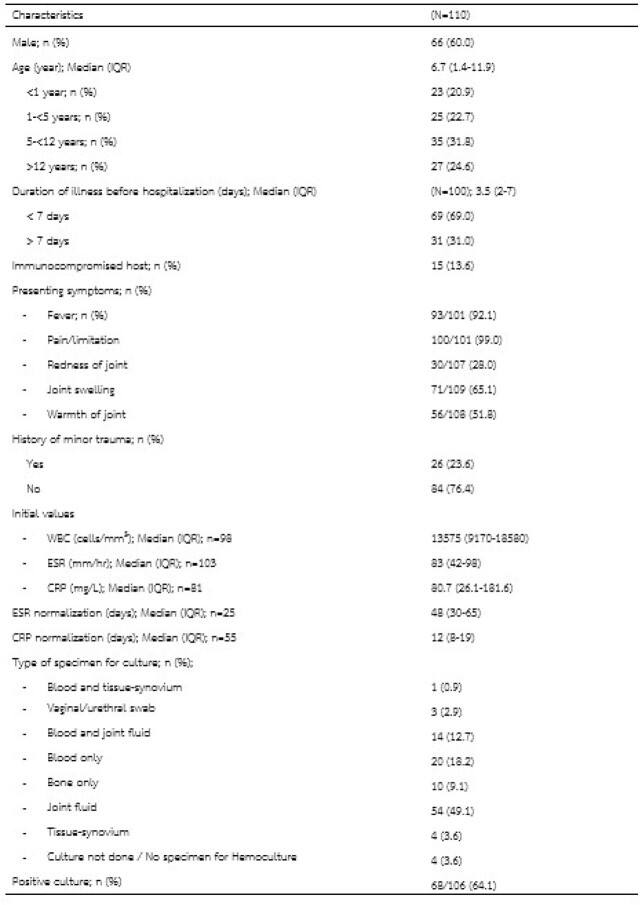

Pathogens according to age groups

**Figure:**
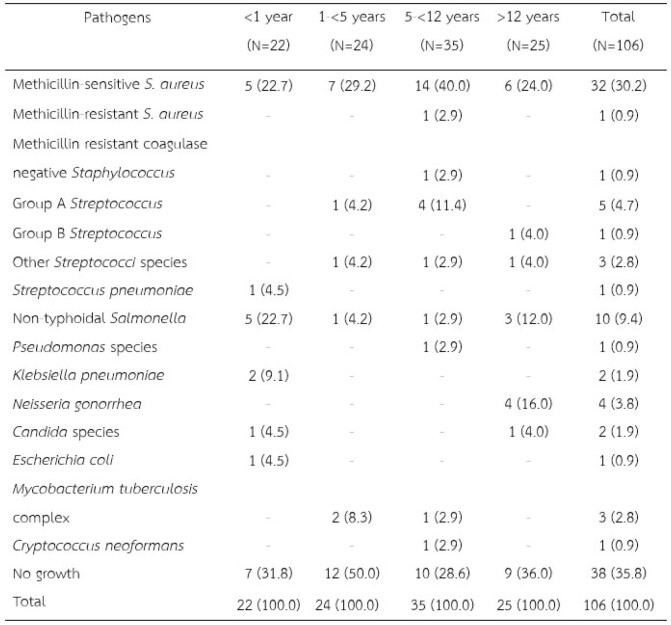

Factors associated with sequalae

**Figure:**
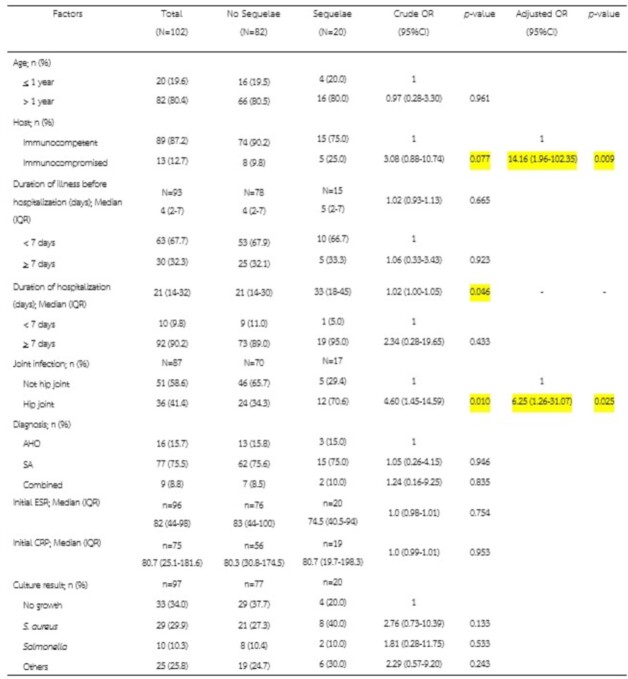

**Conclusion:**

Methicillin-sensitive *S. aureus* was the most common pathogen among Thai children with acute hematogenous osteoarticular infections. However, *Salmonella* was the predominant causative organism among infants. Appropriate empirical antibiotic therapy should be effective against both pathogens in infants. Long-term follow up is needed particularly in immunocompromised children and infection of the hip.

**Disclosures:**

**All Authors**: No reported disclosures

